# PAINLESS: protocol for a triple-blinded randomized sham-controlled multicenter trial of home-based transcranial electrical stimulation for pain management in patients with cancer

**DOI:** 10.1186/s12885-025-15143-3

**Published:** 2025-11-03

**Authors:** Andrea Antal, Perianen Ramasawmy, Lara Rubal-Otero, Ivan Chakalov, Margarita Álvarez Rodríguez, Alexander Hunold, Klaus Schellhorn, María Teresa Carrillo-de-la-Peña

**Affiliations:** 1 Non-Invasive Brain Stimulation Lab, Department of Neurology, University Medical Center Göttingen, Georg-August University, Robert Koch-Straße 40, Göttingen, 37075 Germany; 2 Brain and Pain Lab, Department of Clinical Psychology and Psychobiology, Universidade de Santiago de Compostela, Santiago de Compostela, Spain; 3 Foundation for Health Research Institute of Santiago de Compostela, Santiago de Compostela, Spain; 4 Department of Anesthesiology, University Medical Center Göttingen, Georg-August University, Göttingen, Germany; 5 Fundación Pública Galega de Investigación Biomédica Galicia Sur, Vigo, Spain; 6 NeuroConn GmbH, Ilmenau, Germany; 7 Institute of Biomedical Engineering and Informatics, Technische Universität Ilmenau, Ilmenau, Germany

**Keywords:** Cancer pain, Transcranial direct current stimulation, Transcranial alternating current stimulation, Electroencephalography, Quantitative sensory testing, Non-invasive brain stimulation

## Abstract

**Background:**

Pain is a common debilitating symptom in cancer patients and its management remains a challenge. Among the non-pharmacological analgesic treatment options, low intensity transcranial electrical stimulation (tES) represents a new unique analgesic modality. However, the evidence of tES effectiveness for cancer pain is limited, while the mechanisms of pain relief due to tES are still poorly understood. We propose to test the efficacy of repetitive transcranial direct current stimulation (tDCS) and alternating current stimulation (tACS) in cancer pain patients, and attempt to understand their mechanisms using electroencephalography (EEG) and quantitative sensory testing (QST) measures.

**Methods:**

This article describes the protocol of a multicenter, sham-controlled, parallel-arm, triple-blinded randomized clinical trial assessing home-based tDCS and tACS in the treatment of cancer patients with persistent pain. 450 patients between 18 and 75 years old will be enrolled in this study. Treatment consists of 15 consecutive daily sessions of either anodal tDCS at 2 mA targeting the primary motor cortex or 10 Hz tACS targeting the dorsolateral prefrontal cortex. Following randomization (2:2:1 ratio), 180 patients will receive active tDCS, 180 patients active tACS, and 90 patients sham stimulation. The primary outcome is self-reported pain intensity on a numerical rating scale (NRS) assessed daily (15 days pre-treatment, 15 days during treatment and 15 days post-treatment). The secondary endpoints are medication intake, other cancer-associated symptoms and quality of life. We will also analyze psychophysical and neurophysiological correlates of ascending and descending pain processing using QST and EEG paradigms. Besides the NRS, which will be reported daily, assessments will be conducted at baseline (T0) and at three time points post-intervention (T1, T2, and T3).

**Discussion:**

Positive findings of the study will indicate the therapeutic benefit of tES in patients with cancer-related pain. Group differences in mechanistic measures would yield potential biomarkers for tDCS- and tACS-induced analgesia, which could be used for personalizing and optimizing the intervention. In addition, changes in EEG or QST could provide insights into the mechanisms underlying the tES effects.

**Trial registration:**

The study was registered at the German Clinical Trials Register under the identification number DRKS00031070.

## Introduction

Cancer-related pain—caused by tissue damage due to the cancer itself, metastases, or treatment—is one of the most common and most feared symptoms in patients with cancer [1–3]. Although the prevalence of pain in cancer patients has decreased in the last decade, it still ranges at high levels prevalence of cancer pain is still high, affecting 44.5% of the patients [4]. Cancer pain can persist for or emerge after months or years following treatment [5] and poses a significant burden on patients by impairing their quality of life, daily functioning, and social interactions [5]. Pain has been identified as one of the prognoses for shorter survival in patients, underscoring the necessity to better treat chronic cancer-related pain [6]. Despite the remarkable improvement in the detection and survival rate among different types of cancer patients in the last decades, cancer pain management remains a clinical challenge.

Until recently, the treatment of cancer pain involved primarily analgesic medication, with opioids being the mainstay [7], frequently leading to medication-abuse, dependence, and addiction [8, 9]. To achieve a more sustainable and personalized cancer pain management, the European Society for Medical Oncology recommends a multimodal treatment approach comprising primary antitumor therapy, analgesic treatment, and non-pharmacological and non-invasive therapeutic approaches [10].

Low intensity non-invasive transcranial electric stimulation (tES), especially repeatedly applied anodal transcranial direct current stimulation (tDCS) over the primary motor cortex (M1), has been successfully administered to treat chronic pain across a range of medical conditions, including fibromyalgia, chronic back pain, and neuropathic pain, among others [11–13]. tES, mainly comprising tDCS and transcranial alternating current stimulation (tACS), involves the application of a weak electrical current (1–2 mA) to the brain via surface electrodes placed on the scalp. Unlike tDCS, which involves a direct current with the ability to alter cortical excitability [14, 15], tACS entrains neuronal spiking activity by synchronizing the neural oscillations with the phase of the electrical stimulation through the application of a sinusoidal alternating current [16–18].

Compared to the array of guidelines and reviews on tES in other chronic pain conditions, those addressing the effectiveness of tDCS in patients with persistent cancer-related pain are limited [19–21]. Patients with hepatocellular carcinoma receiving 10 anodal M1-tDCS sessions reported reduced pain and depressive symptoms, with improvements lasting up to one month post-treatment compared to sham tDCS [22]. In patients with advanced head and neck cancer, 20 anodal M1-tDCS sessions significantly produced power spectra changes in several electroencephalography (EEG) bands immediately post-intervention, and pain relief associated with an attenuation of dysphagia and weight loss symptoms usually derived from chemoradiotherapy [23]. In an RCT, patients with primary malignant lung disease were treated with tDCS after going through a thoracotomy, apart from the patient-controlled intravenous morphine system [24]. The authors observed that patients treated with anodal tDCS consumed significantly less morphine during the post-thoracotomy period, compared to those who received sham stimulation. In a recent RCT, female patients with post-mastectomy pain syndrome received 5 sessions of anodal tDCS over M1 for 20 min on each side of the cerebral hemisphere, with those in the active group exhibiting reduced pain severity compared to the sham group [25]. The latest systematic review and meta-analysis on tDCS in pain management in cancer patients showed that anodal tDCS over the M1, left DLPFC, or insula area seems to be effective in reducing pain intensity in cancer patients undergoing surgery, compared to sham, but not in non-surgical patients [21]. Furthermore, tDCS seems to be more effective in pain relief in patients with greater pain intensity and in the long term. However, it is important to highlight that only six studies were included in the meta-analysis. Currently, a bi-center RCT is being conducted to test the effects of five sessions of anodal M1-tDCS on pain and quality of life in cancer patients receiving palliative care [26].

Until now, no study has explored the analgesic effects of repeated tACS in cancer patients with pain and only a few have tested the method in other pain conditions [27]. Ten sessions of 50 Hz tACS at 1 mA applied over the left M1 showed no pain reduction in fibromyalgia patients compared to sham [28]. We previously demonstrated that a single session of 140 Hz tACS delivered at 0.4 Hz over the visual cortex in migraine patients significantly lower pain severity in patients compared to sham tACS [29]. There is also preliminary evidence that a single session of 10 Hz tACS over the dorsolateral prefrontal cortex (DLPFC) delivered at 1 mA for 40 min can reduce pain [30, 31]. Nevertheless, the tES parameters are more heterogeneous for tACS across studies, in contrast to tDCS.

Home-based tDCS under remote supervision following adequate training in use of the technology, either self-administered or delivered by a caregiver, is feasible and safe in different patient populations, including knee osteoarthritis, fibromyalgia, migraine, and psychiatry disorders [32–38]. Thus, our study involves self-administered and remotely supervised at-home tES interventions, rather than clinic-based treatment to make the treatment more accessible to patients in terms of mobility. Quantitative sensory testing (QST) is a widely used psychophysical sensitivity test in clinical research, which assesses the patient’s perceptual response to calibrated stimuli and provides information about underlying mechanisms contributing to the pain sensation [39], having been validated across many chronic pain conditions [40]. Patients with cancer pain exhibit altered thermal detection and thermal pain thresholds compared to healthy controls [41]. Conditioned pain modulation (CPM) [42] and offset analgesia (OA) [43] paradigms, two measures of descending pain inhibition having distinctive mechanisms, have not yet been studied in cancer pain patients using QST. Complementarily, the temporal summation of pain (TSP) paradigm provides insights about pro-nociceptive mechanisms. These measures are included in our study protocol. In addition, we will also measure resting-state EEG and contact heat-evoked potentials (CHEPs), which is an established objective assessment of the function of Aδ and C fibers [44].

Our current multicenter RCT involving 450 cancer patients will be the largest in the literature to test the therapeutic efficacy of home-based tES, especially including both tDCS and tACS delivered for 15 consecutive days. The tDCS arm will receive anodal tDCS over M1. The tACS arm will receive active tACS at 10 Hz over the DLPFC and the control arm will receive sham stimulation (with either the tDCS or tACS montage). Patients and investigators will be blinded to stimulation type. Our primary aim is to test the impact of tES on pain relief and on the improvement of secondary symptoms and quality of life. Our secondary objective is to point out potential mechanisms underlying tES-mediated analgesia in cancer pain using concurrent EEG and QST. We also aim to identify and characterize responders to the intervention based on clinical variables and neurophysiological biomarkers.

## Materials and methods

### Study design

The PAINLESS study, funded by Horizon Europe (call HORIZON-HLTH-2021-DISEASE-04; Project ID: 101047367), is an international multicenter, randomized, placebo-controlled, triple-blind, three-armed clinical trial to test the efficacy of 15 sessions of home-based tES in patients with chronic cancer-related pain. We will explore the effects of anodal tDCS of the left M1 and 10 Hz tACS over the DLPFC on clinical pain, quality of life, cognitive and emotional functioning, fatigue, sleep quality, as well as psychophysical and neurophysiological markers of central pain processing using QST and EEG respectively. Measurement visits will take place at baseline (T0), post-tES intervention (T1), and three months (T2) following the intervention. At the last visit, six months (T3) following the intervention, only the NRS of intensity, unpleasantness and interference of cancer pain with daily life, as well as the NRS of fatigue and sleep quality, will be assessed.

A randomization list is created for an independent researcher for all the clinical sites involved. The study flowchart is described below and summarized in Table 1.

**Table 1:** Study protocol

		Base line	15-days tES intervention		Follow up		
Study activities	Screening Visit 1	Visit 2 (T0)	Visit 3 (Session 1)	Sessions 2–15	Visit 4 (T1)	3-months follow up Visit 5 (T2)	6-months follow up Visit 6 (T3)
Location of meeting	Home or Clinic	Clinic	Clinic	Home	Clinic	Clinic	Home
Screening	✔						
Informed consent and anamnesis	✔						
Baseline questionnaires		✔					
Clinical Interview and History	✔	✔			✔		
NRS pain assessment		Pain intensity rated daily on NRS including 15 days baseline recording				✔	✔
tES training for home stimulation			✔				
Battery of questionnaires		✔			✔	✔	✔
QST-EEG		✔			✔	✔	
Medication diary			✔	✔	✔	✔	
Side/adverse effects questionnaire			✔	✔	✔		
Home-based feasibility questionnaire					✔		
Blinding and satisfactory questions					✔	✔	✔

*tES* transcranial electrical stimulation, *NRS* numerical rating scale, *QST* quantitative sensory testing, *EEG* electroencephalography.

#### Visit 1: screening visit (Study site)

In an interview conducted by the study clinician and a co-investigator responsible for the study, the medical and social background of the participant will be explored with an emphasis on any apparent exclusion criteria. A brief medical screening will be conducted based on the medical history of the patient. During the conversation, patients will be informed about the objective and purpose of the study, including any potential risks of the investigation. The consent form for participation in the study will be signed after the participants and the study clinician are certain of having made an informed decision and having obtained a full case history. During informed consent, the patients’ authorization to access data from their previous clinic records will be requested. It is also an option for each participant to involve a caregiver of theirs, who will help to conduct the home-based tES sessions and provide answers to some questionnaires. The caregivers will also have to sign the informed consent.

#### Visit 2. Baseline assessment (Study site; T0)

During the interview, sociodemographic (e.g., sex, age, lifestyle, medication pattern) and clinical data (comorbidities, type of cancer, extension, time since diagnosis, evolution, previous chronic diseases, risk factors for cancer, antecedents of cancer) will be collected.Patients will also fill in a battery of questionnaires on the web-based online PAINLESS platform (refer to Sect. 5.1 for further details) to assess pain and related symptoms (sleep quality, quality of life, fatigue…) and will take part in the QST and EEG measures. To avoid long assessment periods at the study site, participants will be offered the option to be interviewed online, via a secured videoconference platform and to fill in the questionnaires from home. Moreover, the relatives or caregivers will complete questionnaires addressing their quality of life, the burden of caring for the patients, and their socio-economic backgrounds. Following Visit 2, the patients will be randomly allocated to one of the three intervention groups, using the previously created randomization list. Both patients and clinicians/researchers responsible for the delivery of tES will be blinded to the treatment condition.

#### Visit 3. First tES session and training (Study site)

The first stimulation session will take place in the clinic, supervised by the investigator or clinical staff at the study site. This session also aims to train the patient and/or caregiver to operate and use the tES setup. Participants will receive the tES kit for home use. Further details on the training are found in Sect. 3. Before starting the tES intervention, the patients will complete the daily NRS addressing core symptoms and note medication intake and dosage in a medication diary during 15 days prior to the stimulation session. In addition, during the 15-day period prior to neurostimulation, patients will respond to two questionnaires created ad-hoc for this project, one to assess cognitive complaints and the other to assess socio-economic variables (medical and non-medical) related to the cancer care. The questionnaires will be filled in on the PAINLESS platform.

#### Sessions 2–15. tES intervention (Home-based).

Participants will conduct the tES at home and will complete the assessment questionnaires daily, in the same way as in the pre-treatment baseline period. Besides, during the intervention, participants will report the occurrence of any side or adverse effects of the stimulation.

#### Visit 4. Post-treatment assessment (Study site; T1).

The investigator will interview the patients to assess medication intake, receipt of any non-pharmacological treatment, and number of hours slept in the 24 h prior to the visit. The battery of questionnaires for patients and caregivers as well as the QST and EEG measures for the patients will be conducted, as in Visit 2. Again, the patients will be asked to fill in NRS of the primary and secondary endpoints during 15 days after the treatment. Patients will also fill in the questionnaire on cognitive complaints as in the pre-treatment and report global ratings of improvement and satisfaction with the home-based tES intervention.

#### Visit 5. Three-month follow-up (Study site; T2)

The same procedure as in Visit 4, including QST and EEG assessments will be conducted three-months following the tES intervention. Caregivers will fill in their respective set of questionnaires. The socio-economic questionnaire will also be administered to both patients and relatives/caregivers. Again, we will ask the participants to fill in NRS of the primary and secondary endpoints during 15 days in this follow-up period.

#### Visit 6. Six-month follow-up (Telephone or online; T3)

Six months after the tES intervention, investigators will call the patients via telephone or online to measure the core clinical outcomes on an NRS scale with a one-week recall period. No QST or EEG will be performed. Caregivers will fill in their respective set of questionnaires. The socio-economic questionnaire and a satisfaction survey will also be administered to both patients and relatives/caregivers.

### Specific aims and hypotheses

This multicenter study will address many aspects of cancer-related persistent pain including its potential treatment with repeated tES and assessment of the effectiveness of the therapy using self-reported assessment of different symptoms of the disease, psychophysical testing using QST and neurophysiological testing using EEG. The aims and hypotheses of the study are as follows:

- Aim 1: To determine the therapeutic effects of 15-day home-based anodal tDCS and tACS interventions (compared to sham stimulation) on pain intensity (primary outcome), other dimensions of pain (pain unpleasantness, catastrophizing, interference), quality of life, cancer-associated symptoms (fatigue, depression, anxiety, sleep fragmentation, cognitive impairment), and global functioning among patients with cancer-related persistent pain. We hypothesize that compared to sham, tDCS and tACS will reduce clinical pain and associated symptoms as well as improve quality of life and global functioning. We expect that anodal tDCS might have a stronger effect on pain intensity, while tACS can have a stronger effect of decreasing stress, emotional processing of pain and improving quality of life. We will also test the feasibility of the home-administered tES (analyzing dropout rates, satisfaction scores, adverse and side-effects…). The potential long-term benefits of the interventions will also be addressed at three and six months post-treatment follow-ups.
- Aim 2: To identify psychophysical and neurophysiological predictors and mediators of tES-induced analgesia in patients with cancer pain. We hypothesize that the improvement in clinical pain will be mediated by intervention-induced adaptive changes in (i) nociceptive and pain modulatory system using QST indices and through contact heat-evoked potentials (CHEPs) using concurrent heat painful stimuli and EEG, as well as (ii) in oscillatory activity measured by resting state EEG.
- Aim 3: To identify responders to the tES interventions based on clinically relevant reduction in pain intensity (≥ 30%) and to characterize them according to central pain biomarkers using QST, EEG measures and/or sociodemographic, climical and disease related data. We hypothesize that indices of pain modulation and perception will be sensitive to predict treatment outcomes.

### Study sites and coordination across sites

This clinical study will be conducted at seven study sites, comprising of research centers and hospitals, located in five countries. The study sites are the Servizo Galego de Saúde – Fundación Instituto de Investigación Sanitaria de Santiago de Compostela (SERGAS-FIDIS), Servizo Galego de Saúde - Fundación Pública Galega de Investigación Biomédica Galicia Sur (SERGAS-FBGS), and Universidade de Santiago de Compostela (USC) located in Spain; the Israel Institute of Technology (TECHNION) in Israel; the Universitätsmedizin Göttingen (UMG) in Germany; and the Ente Ospedaliero Cantonale (EOC) in Switzerland. The centers have been selected based on their expertise in either chronic pain, cancer, QST, EEG, and/or neuromodulation. The trial is registered at the German Clinical Trial Register (www.drks.de; ID: DRKS00031070). A lead study coordinator responsible for the administration of the protocol will be assigned at each study site. All the study sites will use identical study methods as described in the protocol, as the staff involved has been trained in on-site and online sessions. The questionnaires and instructions to patients will be provided in the local language of the patients depending on their nationality (Spain- Spanish; Germany- German; Israel- Hebrew; Switzerland- Italian).

The study will be led by the UMG team and the final analysis will be conducted by the USC. The study will use a centralized data management system and a centralized document library (PAINLESS platform; refer to Sect. 5.1. for further details). The PAINLESS consortium hosts monthly videoconferences attended by the study personnel to promote consistent and open communication within the consortium. These meetings are included to ensure consistency among study sites and timely discuss questions or concerns arising during the study course.

### Participants

The PAINLESS study aims to include 450 patients with cancer pain. A relative or caregiver of each patient will participate in the assessments. Each study site will recruit the following number of participants: SERGAS-FIDIS (*n* = 70), SERGAS-FBGS (*n* = 70), USC (*n* = 90), TECHNION (*n* = 100), UMG (*n* = 90), and EOC (*n* = 50) to meet the goal of 450 participants. The underperformance of individual clinical sites in recruitment will be equally distributed by the remaining participating centers until the estimated number of participants is reached. Recruitment will be performed over two years in the involved centers and there will be no competition between the clinical sites.

Each study site will follow a multimodal recruitment strategy involving both clinic-based and community-based methods, tailored to meet the recruitment needs of each study site. Participants will be ambulatory patients receiving care in oncology services, pain units of hospitals, or palliative care services in the respective country. Advertisements through recruitment flyers, letters, information talks for patients, and newspaper articles will be made in hospitals, local oncological practices, and self-help groups for cancer patients. In case the desired number of participants is not reached through these channels for each study site, national associations of cancer patients may be contacted to increase enrolment. Efforts will be titrated to maintain a consistent flow of participants and avoid long waiting times.

Upon interest in participation, the patient will first contact the local study team by e-mail or telephone call. A short pre-screening phone call will be done to inform the patients about the general aspects of the study (duration, place, frequency of visits, commitments), and an appointment will be scheduled for the first visit. At the first visit, the medical doctor or researcher will review the clinical history of the patients to select those that fulfil the inclusion criteria.

We designed the inclusion and exclusion criteria of the study to facilitate the enrolment of a representative sample of cancer patients with chronic pain, whilst optimizing the validity of our study findings and participant safety. Only participants who fulfill all the inclusion criteria and do not meet any of the exclusion criteria will be included in the study. Participants are allowed to continue their treatment-as-usual routine, including medication and non-pharmacological therapies.

Inclusion criteria:

- Diagnosis of cancer affecting the lung, breast, pancreas, or colon (with/-out metastases, except for metastases in the central nervous system).
- Patients aged ≥ 18 years and ≤ 75 years.
- Cancer-related pain must be present for over 1 month prior to enrolment, with a pain intensity rated ≥ 5 on a 0–10 Numeric Rating Scale (NRS).
- Ability to provide informed consent.
- Ability to self-report pain.
- Life expectancy of ≥ 6 months.
- Ability to use the Internet and WhatsApp (Meta, California, USA, etc.) or have a person (relative or caregiver) to support them.

Exclusion criteria:

- Pregnant women or women of fertile age who did not have efficacious contraception during the whole period of the study.
- 45 kg < Body weight < 120 kg.
- Neurological or psychiatric diseases including epilepsy (except anxiety or depression).
- Unstable medical conditions (e.g., uncontrolled diabetes, uncompensated cardiac issues, heart failure, or chronic obstructive pulmonary disease).
- History of neurosurgery, traumatic brain injury with loss of consciousness, and/or cortical lesion. History of unexplained or repeated loss of consciousness.
- History of non-malignant chronic pain.
- Presence of implanted metallic devices in head, neck or chest, as this is a contraindication for tES.

Caregivers of patients should be consenting adults ≥ 18 years old cohabiting with the participants and able to navigate the Internet and WhatsApp.

Participants may decline to participate at any study phase, without suffering any negative consequence. They will be asked to state their reasons for dropping out for monitoring purposes.

### Randomization and blinding

Following the informed consent and baseline evaluations, participants at each center will be locally randomized to the tDCS, tACS and placebo groups in a 2:2:1 allocation ratio, giving more weight to the active interventions. A staff member who is not involved in any study procedures will be responsible for creating the randomization list, generated by an automatic web-based randomization program from GraphPad Prism, and monitoring it during the study. The number of participants in the sham group was planned as half of those receiving tDCS or tACS. This 20% sham allocation was decided upon on account of it being the smallest and statistically correct number of patients that can stay without treatment. Given prior studies in populations with chronic pain, which showed clinically relevant remission of symptoms after tES treatments, we expect a positive effect of the technique in our trial [11]. The corresponding 20% of the sample is sufficient to achieve an analysis with sufficient statistical power. Therefore, for ethical reasons, we are keeping the size of the untreated group to a necessary minimum given our sample size.

The study will be triple-blinded, as the patient and caregiver, the study clinician and/or interventionist, and the evaluator will be blinded to the group assignment until the completion of the trial. The NeuroConn Data analysis will also be conducted in a blinded manner. The device used for the tES intervention (DC-STIMULATOR MOBILE, neuroConn GmbH, Ilmenau, Germany) allows complete blindness. Only the research members in charge of generating the randomization list will have access to the patient’s intervention status. The success of blinding will be measured at the end of the intervention by asking the patients what type of stimulation they think they received.

Unblinding will take place once recruitment and subsequent data analysis are completed. However, the coding system includes a mechanism that allows rapid identification of the treatment procedure in a medical emergency such as (1) in case of administration of an incorrect stimulation dose that could endanger the patient; (2) cases in which further treatment of the patient depends on knowledge of the treatment provided by the medical device;, and (3) in case of serious adverse events.

### Interventionist and assessor training

Study staff and investigators received intensive training in the methods through specialized training workshops (both on-site and online) focusing on the home-based application of tES and use of QST and EEG systems to ensure standard protocols across study sites. Training materials include videos and written manuals for study staff, as well as for patients and caregivers.

## Transcranial electrical stimulation treatment

Home-based tES will be applied during 20 min per session for 15 consecutive days via a DC-STIMULATOR MOBILE stimulator (neuroConn GmbH, Ilmenau, Germany) with a proprietary neuroConn headgear (Knitted tES-CAP, neuroConn GmbH, Ilmenau, Germany) comprising two rectangular textile electrodes of 35 cm² and 25 cm² for tDCS and tACS montages. The caps are of flexible textile material, in five sizes matched different head shapes with respect to circumference and height, and include ear openings and a Velcro chin strap to ensure a comfortable fit with adequate contact between the scalp and the electrodes [45, 46]. The textile electrodes comprise three layers surrounded by diffusion barrier of silicon yarn. The knitted fabric of the bottom layer contains a conductive silicone thread. The middle layer is formed by sponge material providing an electrolyte reservoir for 0.9% isotonic saline solution. The top layer of the cap knitted fabric holds sockets and studs of snap fasteners and red or blue-colored threads indicating the anode and the cathode respectively. Electrodes maintained contact with the scalp via 0.9% isotonic saline-soaked sponges. The press-stud electrode cables clipped on the snap fastener studs in the Knitted tES-CAP connect to the single button-controlled DC-STIMULATOR MOBILE.

The stimulation device is categorized as a Class IIa medical device with manageable risks and approved treatment effects, according to the European guidelines on medical device regulation [47, 48]. All devices will be calibrated, programmed, and checked by the manufacturer, neuroConn GmbH before the start of the study. The pre-programmed tES device stores the patient’s treatment parameters accordingly, to allow for home-based treatment.

*Anodal tDCS* The anode will be placed over the left M1 (C3, according to the 10–20 EEG system) and the cathode will be placed over the right orbito-ocular region (FP2). For the anodal tDCS, a constant current of 2 mA (20 min) with 15 s ramp-up and 15 s ramp-down at the beginning and the end of the stimulation period respectively was applied. The sham tDCS profile will constitute a 15 s direct current ramp-up to 2 mA, 15 s ramp-down to 0.05 Ma, 1130 s of 85 Hz sinusoidal current at 0.05 mA, at the beginning and end of the session, validated in [49]. *tACS* The electrodes will be placed in an F3-F4 montage over the frontal region targeting the DLPFC. A 10 Hz alternating current of 2 mA (peak-to-peak amplitude) will be applied for 20 min, with 15 s ramp-up and 15 s ramp-down. Participants in the sham group will have the same electrode montage as the tES protocols, with a ratio of tDCS to tACS montages 1:1. The sham sham tACS profile will constitute a 15 s 10 Hz sinusoidal alternating current ramp-up to 2 mA, 15 s ramp-down to 0.05 mA, and 1130 s of 85 Hz sinusoidal current at 0.05 mA, at the beginning and end of the session, validated in [50]. *Impedance quality* will be ≤ 15kΩ to ensure proper stimulation of the cortex. The application of tES follows consensus standards in the field, based on previously published guidelines [11, 51].

During each intervention session, participants will be instructed to assume a comfortable position (sitting or lying) and to rest (not to be involved in cognitive tasks or watching TV, but not sleep).

Each participant will receive 15 intervention sessions. The first tES session will take place in the study center (Visit 3), where the patient and/or caregiver will be trained by a researcher and/or clinical staff to operate and use the tES device and accessories. The patient will receive a tutorial handbook with a complete explanation of the procedures for stimulation and will also have access to an online video tutorial in their mother tongue. All supporting material will be available in the native language of the corresponding country. Then, the 14 stimulation sessions (two weeks) will be administered by the caregiver in the patient’s house or self-administered by the patient under remote supervision of the researchers.

The researcher/medical staff will be responsible for remotely authorizing and monitoring the daily stimulation session through the PAINLESS platform. In case of emergency, the patient or caregiver should call the numbers given them to the training session (medical emergency and stimulation related emergency). At the end of the whole treatment (15 days), researchers will be able to see whether the sessions were carried out correctly (day, time, duration and further configuration settings of the stimulator) without affecting the blinding of the study. This information is stored in a log file generated by the stimulator and saved in the storage module of the stimulator. After treatment completion, the log file and the stimulation data (applied current, adjusted voltage and resulting impedance) will be uploaded to the PAINLESS platform. Only authorized research members will be granted access to the stimulation data. Patients will provide their daily NRS and complete the medicine intake diary using the online PAINLESS platform, which will define an additional access in a patient’s role.

## Assessments and measures

### Primary outcome

The primary outcome will be self-reported cancer pain intensity, assessed by using the 11-point NRS [0: no pain due to cancer; 10: intolerable pain due to cancer] [52, 53]. The cancer pain intensity will be reported daily on the PAINLESS platform for 45 days (15 days pre-treatment, 15 days during, and 15 days post-treatment, recorded about the same time of the day). In addition, it will be reported at T0, T1, T2 and T3 visits with a recall period of 1 week. The reproducibility and cross-cultural validity of the NRS have been shown in chronic cancer-related pain, making it a reliable measure for pain assessment as a clinical outcome in many studies [54].

We will follow an intention-to-treat approach with missing data being imputed following the method that allows us to obtain the best adjustment.

### Secondary outcomes

#### Self-reported questionnaires

Patients will fill in a battery of scales and questionnaires at T0, T1 and T2, using the PAINLESS platform. At T3, only some of the scales will be delivered telephonically or online.

1. Pain and its dimensions
  The NRS for pain unpleasantness [0: not unpleasant at all; 10: maximum unpleasantness of pain] will be reported daily 15 days pre-, during, and post-treatment, similar to NRS for pain intensity, and assesses the impact of the intervention on the affective-motivational dimension of pain. NRS will also be assessed at T0, T1, T2, and T3 visits with a one-week recall window. This score has been shown to exhibit good sensitivity as well as discriminant and convergent validity and sensitivity to change [55].
  The NRS for the interference of cancer-related pain in daily life during the last day [0: no interference at all; 10: maximum interference] will be reported daily 15 days pre-, during, and post-treatment, to address the extent to which cancer pain hinders the individual’s engagement with their daily activities.
  The 9-item Brief Pain Inventory (BPI) assesses pain holistically, including the history, location, severity, and impact. The Pain severity (Items 3 to 6) and the Interference components (Item 9) of the BPI will be used as outcome variables [56]. Higher scores indicate higher severity.
  The 13-item Pain Catastrophizing Scale (PCS) tests the level of pain catastrophizing in patients, defined as exaggerated negative cognitive-affective response to actual or anticipated pain experiences [57, 58]. Higher scores reflect higher levels of catastrophic thinking. The overall score will be calculated (range 0–52), with a total score of >30 indicating a clinically significant level of pain catastrophizing. High levels of pain catastrophizing in cancer patients with chronic pain have been associated with reduced quality of life and higher levels of pain, fatigue, and sleep impairment [59].
2. Quality of life
  The total score of the 36-Item Short Form Survey (SF-36) (Ware, 2000), a commonly used and validated questionnaire to assess the quality of life in different populations of cancer patients and across countries [60–63], will be used to determine the quality of life of participants (range 0–100). Higher scores indicate higher quality of life.
  The European Quality of Life 5 Dimensions 3 Level Version (EQ-5D-3 L) instrument [64, 65], a valid and reliable questionnaire in both European and non-European cancer patients [66–68], will also be used to measure health-related quality of life.
  Composite scores of these two questionnaires will be also investigated.
3. Fatigue
  The 21-item Modified Fatigue Impact Scale* (*MFIS) [69, 70] will be administered to assess the level of fatigue, a debilitating consequence of cancer, affecting over half of cancer patients [71, 72]. The scale accounts for the physical, emotional, and social components of fatigue and the overall score ranges from 0 to 84, with higher scores representing higher levels of fatigue.
4. Sleep quality
  Sleep fragmentation is one of the most distressing symptoms impeding the quality of life of cancer patients throughout all stages of the disease and all phases of treatment, with a prevalence of 60.7% of sleep disturbance [73]. The 12-item Sleep Scale from the Medical Outcomes Study (MOS) scale [74], a frequently used subjective assessment of sleep disturbance in patients with malignant or chronic illnesses [75–79], will be used to assess sleep quality of patients. The aggregated MOS score (range 11–71) will be calculated, with higher scores representing greater sleep dysfunction.
5. Performance status
  The 6-point scale Eastern Cooperative Oncology Group (ECOG) scale [80] (0: fully functional and asymptomatic, 1: symptomatic, fully ambulatory, 2: symptomatic, in bed or chair < 50% of the day, 3: symptomatic, in bed or chair >50% of the day, 4: bed- or chair-ridden, 5: dead) will be administered to assess how the patient’s disease is progressing and evaluate how the illness affects their abilities of daily living, including self-care.
6. Psychological impairment
  The 9-item Patient Health Questionnaire (PHQ-9) [81, 82], a self-administered instrument for screening, monitoring, and assessing the severity of depression, will be used to assess the depression level of the participants. Higher scores are associated with higher levels of severity.
  The 7-item Generalized Anxiety Disorder (GAD-7) scale [83] will be implemented to assess the level of anxiety in patients, with higher scores indicating higher levels of anxiety.
7. Cognitive functioning
  Cognitive complaints are one of the most common consequences of cancer and its treatment, and have a negative impact on the well-being of patients. The 20-item *ONCOFOG (OFQ) questionnaire* will be used to assess the cognitive complaints (including different domains such as attention, memory, etc.).
8. Subjective improvement and satisfaction
  The participants will fill in an ad-hoc questionnaire. The instrument will aggregate all the physical and emotional functioning, side effects etc. into an overall measure of their perception of the advantages and disadvantages of the treatment received.
9. Medication diary
  The participants will record their daily use of medication, including type and dose, in a medication diary throughout the study.
10. Socioeconomic status
  A socio-economic variables questionnaire will be administered to explore the financial costs of cancer (direct and indirect, medical and non-medical costs) for patients in home care. Its analysis will enable assessment of the socioeconomic cost/benefit associated with the tES treatment.

The following questionnaires will be completed on the PAINLESS platform by the relative/caregiver in charge of assisting the patient in day-to-day life and supporting the patient in conducting the home-based tES:

- The 12-item Zarit Burden Interview (ZBI), a specific instrument for the relatives/caregivers of cancer patients will be used to assess the burden (stress and negative feelings) associated with the caring task [84].
- The SF-36 will be filled in to assess the impact of the intervention on the quality of life of care providers [85]. This is also an indirect measure of the patient’s autonomy.
- A socio-economic variables questionnaire to explore the economic costs associated with ambulatory and home-based care for patients with cancer. The analysis of this questionnaire will serve to assess the costs and benefits associated with the tES intervention, from the side of the relatives.

#### Psychophysical and neurophysiological measures

##### Quantitative sensory testing

Graded thermal stimulations will be applied to the skin by the TCS II thermal contact stimulator (QST.Lab, Strasbourg, France), which comprises several micro-Peltier thermo-electric elements that achieve very steep temperature ramps (i.e., up to 300 °C/s). The thermode has a total stimulation surface of 4.5 cm^2^. That way, the time required to estimate the pain threshold with this method is less than 2 minutes per skin area. We can, thus, easily activate small, thermal nociceptive nerve fibers conveyed by the spinothalamic tract. These properties yield a cost-effective test with reduced test-retest variability, fewer safety precautions, and a lower burn risk than conventional laser stimulators. The perceived pain intensity of ongoing thermal stimulations will be assessed using an 11-point NRS (0: no pain; 10: worst pain imaginable). The patients will be seated during the experiments, with eyes open. Room temperature will be controlled.

**Fig. 1:**
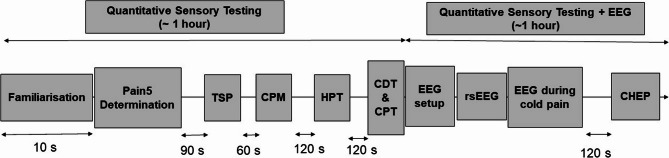
Timeline for each assessment visit including measures of quantitative sensory testing and electroencephalography. TSP, Temporal Summation of Pain; CPM, Conditioned Pain Modulation; HPT, Heat Pain Threshold; CDT, Cold Detection Threshold; CPT, Cold Pain Threshold; OA, Offset Analgesia; EEG, electroencephalography; rsEEG, resting state EEG; CHEP, Contact Heat Evoked Potential

The QST procedure is detailed below in order of application and summarized in Fig. 1.

1. Familiarization
  Participants undergo a short training to familiarize themselves with the heat stimuli.
  a Heat pain: 1 stimulus of 45 °C for 5s. Ramp-up and ramp-down: 170°C/s.
  b Cold pain: 1 stimulus of 0 °C for 5s. Ramp-up and ramp-down: 170°C/s.
2. Pain5 determination
  Pain5 is defined as the temperature resulting in pain intensity ratings of 5 on the NRS scale, which will be determined for the temporal summation of pain (TSP) and the conditional pain modulation/offset analgesia (CPM/OA) procedures separately. The determination process will be repeated twice for each. The mean of the temperature yielding a pain intensity self-report of 5 will be calculated for each of the TSP and CPM/OA determinations. For determining the Pain5 for both TSP and CPM/OA, the starting temperature is set at 45 °C. If the pain intensity rating is > 5/10, we will reduce the temperature by −1 °C (i.e., 44 °C) for the following stimulation. Conversely, if the pain intensity rating is lower than 5/10, we will increase the temperature by + 1 °C (i.e., 46 °C) for the following stimulation. This process is repeated until an NRS of 5 is achieved. The inter-stimulus interval is 5s. The difference in the procedures is a stimulation duration of 0.5 s and 10 s for TSP and CPM/OA respectively.
3. Temporal summation of pain
  TSP refers to the increased pain perception from repetitive noxious stimuli resulting from wind-up, whereby spinal neurons exhibit enhanced firing owing to repetitive input to the C-fibers [86, 87]. The test-retest reliability of thermal TSP has been previously validated, supporting its implementation as a consistent behavioral correlate of central sensitization [88, 89]. Initially, a single stimulus at the determined Pain5 for TSP is applied, with a pulse duration of 0.5s and a ramp-up and -down of 170 °C/s in the dominant volar forearm. The participant is asked to rate the pain intensity for this stimulus, with a maximum of 10 s to rate the pain. Then, 10 stimuli at the determined Pain5 for TSP will be applied with a pulse duration of 0.5s, a ramp up and down of 170 °C/s, and an inter-stimulus interval of 0.5s (i.e., stimulus onset to onset interval of 1 s and stimulation frequency of 1 Hz). The participant is asked to rate the pain intensity for the last of the stimuli in the sequence. The TSP index will be calculated as the normalized difference in pain intensity ratings between the initial single stimulus and the last pulse of the 10-stimulus sequence [90].
4. Conditioned pain modulation
  CPM, known as the ‘pain inhibits pain’ phenomenon, appears when the presence of a second noxious stimulus (conditioning stimulus) reduces the pain perceived from an initial noxious stimulus (test stimulus) [91]. CPM has been described as the behavioral correlate of diffuse noxious inhibitory control, a phenomenon whereby inputs from peripheral C-fibers lead to diffuse inhibition from the brainstem of incoming noxious stimuli mediated by heterotopic C-fibers [42, 91, 92]. First, the test stimulus at Pain5 for CPM/OA is applied for 10 s, with a ramp-up and -down of 170 °C/s in the dominant volar forearm. The participant is then requested to rate the pain intensity perceived. Subsequently, the participant is asked to place the palm of their non-dominant hand on the cold plate. After 20 s, they are asked to rate their perceived pain. At 30 s after placing the palm on the cold plate, the test stimulus is applied again and the pain rating is measured. As soon as the evoked pain intensity for the second test stimulus application (conditioning stimulus) has been produced, the palm of the non-dominant hand is removed from the cold plate. The stimulation site for applying the test stimulus will be slightly moved for each application to avoid thermal receptor habituation. The CPM index is calculated as the normalized difference between the evoked pain ratings for the test stimulus before and after the application of the conditioning stimulus [93].
5. Thermal pain and detection thresholds
  The heat pain threshold (HPT), the cold detection threshold (CDT), and the cold pain threshold (CPT) will be determined using the previously described methods of limits [39]. For each threshold, three trials will be performed with 5 s inter-trial intervals. For the HPT and CPT, the participant will be asked to press the response button as soon as a hotness or coldness perception becomes painful respectively. For the CDT estimation, the participant is asked to press the response button as soon as coldness is perceived. The stimulation site will be the dominant volar forearm and will be moved slightly between each stimulation to avoid habituation of the thermal receptors. Temperature will change (i.e., increases for HPT, and decreases for CDT and CPT) at a rate of 1 °C/s. Between HPT and CPT trials (i.e., as soon as the response button is pressed) temperature returns to the baseline level at a rate of 8 °C/s, while for CDT it returns at a rate of 170 °C/s. To determine the threshold, the arithmetic means of the temperature at the response button press across the three trials for each threshold will be used as the corresponding threshold estimation.
6. Offset analgesia
  OA is defined by a profound decrease in perceived pain intensity on a slight decrease in temperature during noxious thermal stimulation, which is more disproportionate than expected from the rate of temperature reduction [94, 95]. Unlike CPM which relies on solely central mechanisms, OA is mediated by both peripheral and central mechanisms [43]. CPM and OA activate distinct brain areas, engage different inhibitory processes in the central nervous system, and demonstrate no correlation between indices [96]. This protocol will require an electronic visual analogue scale (eVAS 0–10). Heat stimulation will be delivered to the dominant volar forearm. First, a familiarization step will be taken with the stimulation intensity set at the determined Pain5 for CPM/OA minus 2 °C, with a duration of 10s. Participants will be asked to use the eVAS device to rate the perceived pain intensity evoked by this stimulation. OA will be induced by applying the determined Pain5 temperature during 5 s, then Pain5 plus 1 °C for another 5 s, and then again, the Pain5 for 20s. A constant heat stimulus with the pre-determined Pain5 for 30 s will be used as a control stimulus. It will be applied either before or after the OA stimulus according to a counterbalancing scheme. Patients will rate their pain level continuously on the eVAS during both OA and control stimulations. The OA index is calculated as the normalized difference in pain intensity ratings during the last 20 s of the OA trial and the control trial.
  The baseline temperature will be set at 32 °C for Pain5 determination and TSP, CPM, HPT, CDT, CPT, and OA measures.

##### Concurrent EEG-QST measures]

EEG measures will be recorded using a portable, adaptable, and simple-to-use MENTALAB Explore + recording system developed and adapted to clinical use (Mentalab GmbH, Munich, Germany). EEG signals will be acquired by 32 electrodes mounted in an elastic cap and arranged according to the international 10–20 system. The EEG signal will be amplified and digitized using a sampling rate of 250 Hz, and the reference will be placed at CPz. Impedances will be kept under 10kΩ for all electrodes.

Firstly, resting state electroencephalography (rsEEG) will be recorded for 5 min with participants maintaining their eyes closed. The participant will be instructed to sit comfortably and relax letting their mind wander. Next, a second rsEEG recording will be performed, but with a cold noxious stimulus being applied to the palm of the dominant hand. At the end of the test, the participant will be asked to report the average level of pain perceived during the 5 min. The intensity of the cold pain stimulus must be between 3 and 5/10 on the NRS during the whole recording. The timeline for the concurrent measures is also illustrated in Fig. 1.

Finally, we will record Contact Heat Evoked Potentials (CHEPs) using concurrent QST-EEG.

CHEPs, mediated by Aδ fibers, are a neural correlate of nociceptive processing and dysfunction in humans [44, 97, 98]. CHEP parameters can inform researchers about the maladaptive neuroplastic changes in chronic pain patients, though this is not yet characterized in cancer patients [99]. We will apply 25 stimuli at 60 °C with a duration of 0.3 s and ramp-up and -down of 170 °C/sec, with an inter-stimulus interval of 10 s, to minimize saliency and startle responses. EEG will be simultaneously recorded. The baseline temperature will be 32 °C. If the stimulus is very painful, the temperature will be reduced by −1 °C. This process can be repeated until 55 °C is reached. At the end of the test, the participant will rate how painful the stimuli sequence was on average. The heat stimulation will be applied to the dominant volar forearm. The stimulation site will be moved slightly between each stimulus application to avoid habituation of the thermal receptors.

Amplitudes of the N1-P1 complex, N2, and P2 components during hot pain stimulation as well as time-frequency analysis of the EEG signals will be the focus of data processing. Quantitative EEG analytical techniques will enable the evaluation of changes in spectral power, coherence, and phase connectivity.

For the different QST stimulations, if the dominant volar forearm and palm are primary pain sites, the procedure should be applied to the non-dominant arm counterparts. If this is also considered a primary site of pain, the dorsum of the dominant forearm or hand will be used instead.

## Data management and analysis

### Online data management system

The clinical data of the patients will be collected in the web-based PAINLESS platform, which was developed by Software Imaging & Vision information technology company (SIMAVI, Bucharest, Romania). The PAINLESS system is maintained on the infrastructure of the Supercomputing Centre of Galicia along with the associated collected data.

Patient data will be treated as strictly confidential, and the data collection will be pseudo-anonymized, protecting the participants’ identity. For this purpose, the study doctor will assign a unique code called the “Painless ID” to the participant with which they will be identified during all subsequent phases of the study. The data relevant to the study, except for patients’ names, will be recorded, processed, and stored together with this unique code identifier and all the clinical data related to the state of health. The profile data of the patient is stored in a different repository to the clinical data in order to create another layer of security. Only the physician and authorized parties will be able to link this code to the patients’ real identity. The only stored personal data relevant to the study are age and sex and does not identify the patient.

The system automatizes a lot of the processes related to the study. The registration process is automatized through the PAINLESS Platform. There is an Eligibility Questionnaire where the clinician enters the Painless ID and the answers the patient gave to some questions that are important to determine if the patient is eligible to participate in the project. The system then compares the answers to the eligibility criteria determined by the consortium and returns an Eligibility Code only if the patient is declared eligible. The patient can then use the Eligibility Code to register through the platform and will receive an email that they have successfully registered to the Painless Platform. A similar process is performed for caregivers, a caregiver code is generated associated with an existing patient and can be used code to register in the platform. Password recovery can also be done through email. The registration process has multiple steps in order to ensure the Painless Platform is a closed ecosystem and only selected personnel and patients have access for security purposes.

Patients, caregivers and clinicians can complete questionnaires about the state of health of the patients. The questionnaires become unlocked in the platform at different times based on the decisions of the clinicians. After a questionnaire is submitted, the responses are stored in the database of the project along with some calculated scores in the case of some questionnaires. The clinician is able to see how a patient answered their questions on the platform.

Another useful functionality of the PAINLESS platform is the ability to store medical files generated during the PAINLESS project. The project works with 3 different device types that generate files and the files can be saved, stored, visualized, downloaded, and validated through the platform. Some of the data are accessible to clinicians to visualize but some information is is able to be seen only available if to a person designated as administrator in each organization downloads the full file. This is done in order to protect the integrity of the research since most clinicians should not be informed if the patient has received a real tES stimulation or a sham. For QST and EEG, MentaLab type data, the process is the same to provide consistency to the platform.

The patient data are subject to medical confidentiality and the provisions of the General Data Protection Regulation of the European Union. Only information that cannot be identified can be passed on to other members of the consortium. The data will be kept pseudo-anonymized at the end of the study, with the destruction of the code identity relationship for future use, subject to express permission being sought and provided that the participants agree to it. The data will be stored for 15 years and then deleted. Participants have the right to access, to rectify, to erasure, to data portability, to be notified, to object, and the right regarding automated individual decision-making, including profiling.

All the data collected in the PAINLESS project will be used to train artificial intelligence models, but no personal data will be used. The models will have two primary functions: to improve knowledge about palliative care for patients and to increase the standards of care for cancer patients. The patients are informed prior to registering that their data will be used for artificial intelligence, and they must consent for their data to be used. To preserve human agency, the system will not transmit recommendations directly to the patients, all such recommendations will first be checked by a medical professional to ensure their validity.

### Sample size calculation

To calculate the sample size, we considered the data of a recent meta-analysis of 13 studies with data originating from 398 participants with chronic pain. Active tDCS has been reported to have a superior effect than sham with a medium-large size effect (standardized mean difference= −0.76 +/– CI 95% = −1.24) [100]. However, the effect size of tDCS varied considerably between studies, for which reason we decided to achieve small effect sizes to ensure the robustness of our results. Thus, we used G*Power software (Version 3.1.9.7) to calculate the sample size sufficient to reach a small effect size (f = 0.095) with mixed model analysis of variances (ANOVA), setting alpha at 0.05 and power (1-β) at 0.9. The sample was estimated as 291 participants. Thus, we will recruit 450 participants to compensate for eventual dropouts or attrition during the treatment or at the follow-ups (approximately 30%).

### Statistical analysis plan

The statistical plan was accepted by the ethical committees of the involved centers. To ensure that the treatment groups (tDCS, tACS, sham) were comparable, we will perform a one-way analysis of variance (ANOVA) for age, gender, clinical outcomes and a chi-squared analysis for the demographic variables, medication pattern, and the rest of categorical variables.

 Aim 1 and 2: We will implement an intention-to-treat analysis approach, including all randomized participants who received at least 10 consecutive stimulations [101, 102]. Missing data will be imputed following the method that allowed us to obtain the best adjustment. We will address our primary and secondary hypotheses using two complementary statistical approaches (Classical Frequentist and Bayesian statistics) to enhance the robustness of our findings (similar to Samartin-Veiga et al., 2022), as well as time series analysis. We will calculate mixed model ANOVAs for each outcome (scores of self-report questionnaires for both patients and care providers, as detailed in Sect. 4.2; QST and EEG measures) with the intervention group (tDCS, tACS, sham) as a between-subject factor and time (T0, T1, T2, T3) as a within-subject factor. Age, sex, and cancer characteristics (i.e., type extension, evolution, previous treatments) will be included as covariate variables in the statistical analysis. In the presence of statistically significant main effects and group*time interaction effects, Bonferroni-Holm corrected post hoc analyses will be conducted for multiple comparisons. The effect size will be computed as eta squared η2 and interpreted based on Cohen’s benchmark categorization (small, η2 = 0.01; medium, η2 = 0.06 and large, η2 = 0.14) [103]. The normality of residuals will be tested using Shapiro Wilk’s test and normality plots. The homoscedasticity assumption for mixed model ANOVA will be tested with the Levene’s test and the sphericity will be tested using the Mauchly’s test for repeated factors. In case of sphericity deviation, Greenhouse-Geisser corrections will be used.

Aim 3: Patients will be classified as *responders* to the intervention if they report a mean reduction in NRS pain intensity of at least 30% at T1 compared to T0. To characterize the best responders to tES, we will also use the algorithms developed in the precursor study to profile patients using the sociodemographic and clinical variables and biomarkers studied. We will conduct tree-based methods (linear mixed-effects model trees or, in broader terms, generalized linear mixed-effects model trees), which provide a convenient way of visualizing decision rules for predicting an outcome. Personalized treatment methods will be determined by using a convenient categorical tree-based algorithm.

Statistical significance will be set at *p* < 0.05.

### Data sharing and dissemination

The consortium will ensure open access to the methods and data collected (not subject to intellectual property protection) in this multicenter study. To facilitate the replication of results and coordinated data analyses, we will use free software whenever possible. In particular, the code used for stimulation in QST will be written in the open-source software Psychopy and will be made available for anyone to download on GitHub. This open publication of the methods will allow easier replication and collaboration with other researchers. Electrophysiological data will be available for download on an online repository (brainsignals.de, physionet.org). Finally, the code used for data analysis (mainly in Python and Matlab) will also be uploaded to GitHub as Jupyter Notebooks. Jupyter Notebooks are a very interactive and simple way to test and manipulate codes, and they provide the perfect environment for publishing tutorials on data analysis. Open sharing of data, codes, and materials increases reproducibility and transparency, thus addressing an important problem in current neuroscience research using the Zenodo portal developed in the context of OpenAire.

Data sharing inside and outside the PAINLESS consortium is guaranteed through the use of the resources provided by the European Open Science Cloud and the European Data Infrastructure, which offer a virtual environment to store, share, and re-use large datasets and the supercomputing capacity to process them.

## Ethical aspects

### Ethical compliance

This PAINLESS protocol has been approved by the local ethical committees of the USC, SERGAS-FIDIS, SERGAS-FBGS, UMG, and EOC. The study will be conducted under the applicable national, European Union-implemented, and international laws and regulations. All the methodologies will comply with the Declaration of Helsinki and its modifications, the International Council for Harmonization of Technical Requirements for Pharmaceuticals for Human Use – Good clinical practice, the UNESCO Universal Declaration on Bioethics and Human Rights, and the European Medical Device Regulation. Written and verbal information will be provided and informed consent will be obtained from all participants. Participation in the study is voluntary and free. No additional fees will be charged to the participants, and no payments will be made.

### Risk-benefit-assessment

Low-intensity tES may cause minor adverse effects, such as mild headaches and skin irritation on the scalp. Recent studies confirm that tES is completely safe if inclusion and exclusion criteria are met [51]. Burns can occur if liquids other than saline solution are used to wet the electrodes. However, in this study, participants will be provided with sufficient saline solution to use for home-based tES. The home-stimulator device that will be used in the study continuously monitors the impedance of the electrodes, so that the stimulation will be stopped even during the stimulation in case the impedance value increases over the assigned threshold.

The following adverse effects have been observed and documented in the literature [51]:

1. Generally, about half of the subjects feel a tingling or light-burning sensation under the electrodes during stimulation, which depends on the current density.
2. Slight redness of the skin after stimulation, which disappears after 10–30 min is observed in 30–40% of the volunteers.
3. Light headaches after the stimulation can be observed in less than 10% of the volunteers.
4. Light fatigue upon stimulation is observed in less than 10% of the subjects.

Based on the summaries related to clinical conditions and healthy subjects, any form of tES under 60 min (duration) and 4 mA (intensity) is considered to be safe based on the opinion of experts in the field of non-invasive brain stimulation [51].

## Discussion

Persistent cancer-related pain, arising from the disease itself or its treatment, is one of the most debilitating consequences of cancer, with a substantial impact on the daily functioning and quality of life of the patients [7, 104]. Through the PAINLESS multicenter study, we are proposing an easy-to-administer remotely supervised at-home non-pharmacological tES intervention for persistent pain in cancer patients. The findings of the study have the potential to provide a novel avenue to better manage pain in patients with different types of cancer. Despite pain relief and its mechanisms being the main focus of this study, we are also interested in the impact of the therapy on quality of life, emotional and psychological well-being, fatigue, and dependency on caregivers in the patients. Anodal tDCS over M1 has been proven effective in managing chronic pain in different medical conditions such as migraine, neuropathic pain, fibromyalgia, myofascial pain, and postoperative pain, among others [11, 105–107], while the therapeutic potential of 10-Hz tACS is under exploration [27].

With research using low intensity neuromodulation therapies in cancer-related pain still in its infancy, our sham-controlled triple-blinded multicenter study aims to unravel the analgesic potential of repeated anodal tDCS and 10 Hz tACS and to underpin the underlying mechanisms through behavioral and neurophysiological correlates of pain. The cortical effects of tDCS arise from the modulation of neuronal membrane resting potentials, resulting in altered glutamatergic and/or gamma-aminobutyric acidergic neurotransmitter release. N-methyl-D-aspartate receptor-regulated neuroplastic changes in long-term potentiation- or long-term depression-like manner are believed to underlie the long-term effects of tDCS. The net tDCS-induced effects depend on many methodological factors such as the electrode position and the duration, polarity, and intensity of the stimulation [108]. Repeated anodal tDCS of M1 mediates analgesia both through local and network effects in the brain, employing long-lasting neuroplastic modifications that outlast the stimulation period [108–110]. Pain relief depends on the modulation of connections between the M1 and the thalamic nuclei as well as communication with other brain areas—cingulate gyrus, prefrontal cortex, insula—and works as a top-down regulation [107, 111]. The thalamus also potentiates the endogenous descending pain inhibitory system, leading to analgesia [107]. Hence, pain reduction can be observed both on the sensory-discriminative (pain intensity) and affective-motivational (pain unpleasantness, suffering) dimensions of pain.

The only study to have used 10 Hz tACS in chronic pain reported a significant decrease in pain intensity in patients with lower back pain compared to sham [31]. tACS can enhance the functional connectivity between targeted brain areas by entraining brain oscillations synchronously [112]. Since pain is encoded by a temporospatial brain network integrating sensorimotor, cognitive, and emotional-affective dimensions of pain, the application of tACS between targets on each hemisphere might be helpful in pain management by altering pathological oscillatory brain communication [113]. Nevertheless, the exact mechanisms of cancer-related pain remain elusive for both tDCS and tACS.

In this study, we opted for self- or caregiver-administered and remotely supervised home-based tES over daily in-person stimulation at the study sites to improve the accessibility of the intervention to the patients. Persistent pain represents a significant barrier that can affect the ease of travel to health facilities and cause difficulties in accessing healthcare and treatments, with consequent economic and organizational impacts. The mobility limitations faced by chronic pain patients have brought about heightened awareness of the need to develop new health services based on digital technologies [114, 115]. Generally, it has been observed in patients with chronic pain that the improvement of monitoring through a telemedicine-oriented approach capable of guaranteeing continuous and effective communication with health care professionals can lead to a significant increase in the effectiveness of treatments [116]. Through a home-based approach both for tES as well as for daily clinical monitoring, we expect to reduce the direct and indirect healthcare and social costs, while improving the access to healthcare using e-health tools.

Beyond the greater accessibility of the treatment through home-based technology, our RCT overcomes commonly observed methodological limitations in previous studies testing the effectiveness of tES in chronic pain, including cancer-related pain. This study is a multicenter trial with a powered sample of 450 patients, addressing the underpowered sample sizes used in most studies [11, 19–21]. An important strength of our methodology is the study of the potential mechanisms underlying pain relief in cancer pain using QST and EEG measurements, in addition to self-reported questionnaires and ratings of pain and associated symptoms. Around 50% of studies in a systematic review reported sensory abnormities using thermal detection thresholds (CDT and HDT) and abnormal thermal pain processing using thermal pain threshold (CPT and HPT) [41] in cancer pain (mostly chemotherapy-induced neuropathic pain). The not yet studied QST-based conditioned pain modulation, temporal summation of pain and offset analgesia, and EEG-based CHEP measures will shed new light on the pro-nociceptive and anti-nociceptive mechanisms involved in tDCS- and tACS-induced analgesia in cancer pain patients.

The findings of small clinical trials are typically difficult to translate into clinical practice, conducive to outcomes commonly reported as statistical comparisons between the means of treatment groups. Therefore, previously validated responder analyses might provide a reliable and clinically relevant approach in analgesic studies [117, 118]. In our study, we will identify responders based on a clinically relevant pain reduction on the NRS and conduct responder analyses to profile them based on the studied biomarkers. This will aid clinicians in making decisions on whether the tES therapy is likely to work in a patient and, if so, help them to determine the expected degree of pain relief is.

While previous cancer trials study tES in patients with chronic pain arising from a specific cause [19–21], our study is more inclusive, allowing cancer patients with different types of persistent pain ranging from treatment-related pain to neuropathic pain and pain due to cancer itself. The involvement of a heterogeneous patient group aims to provide a more comprehensive picture of the benefits and drawbacks of therapies as they are used in clinical practice. However, the heterogeneity of the etiology of pain across cancer patients poses another challenge in interpreting the mechanisms of tES-induced analgesia. Cancer pain is multifaceted and can be categorized as nociceptive pain, neuropathic pain, and a combination of the prior types [119]. Nociceptive pain is caused by stimulation of nociceptors in the skin and deep musculoskeletal structures, usually proportional to the degree of receptor activation (e.g. tumor invasion into bone, postsurgical pain). Neuropathic pain is due to primary dysfunction or lesion in the peripheral or nervous system [120] and might be untreatable with opioids (e.g. tumor invasion in nerves, chemotherapy-induced neuritis) [119]. Since nociceptive and neuropathic cancer pains exhibit distinct central mechanisms [121], the efficacy of the tES therapy and the QST outcomes might differ. Notwithstanding the strength of a multicenter RCT in validating novel therapeutic interventions across countries, one of its limitations in patients with chronic pain is the diverse viewpoint on pain perception, arising from racial, ethnic, cultural, and national differences. Depending on their ethnic backgrounds, patients might view, rate, and respond to pain differently [122]. It has been shown that pain activates stress-related physiological responses across groups differentially and that different groups use different coping strategies, with the mechanisms underlying the discrepancies in pain response being complex and multifactorial [123].

In conclusion, this multicenter study will elucidate the potential of anodal M1-tDCS and 10 Hz tACS in reducing pain and improving quality of life and function in cancer patients with persistent pain. The systematic prospective appraisal of the response to treatment, the assessment of the feasibility of home-based stimulation, the identification of responders, and the characterization of mechanistic pain-related biomarkers will contribute to advancing the optimization and personalization of tES in chronic pain and oncological research.

## Data Availability

No datasets were generated or analysed during the current study.

## Abbreviations

ANOVA: Analysis of variance
BPI: Brief Pain Inventory
CDT: Cold detection threshold
CHEP: Contact heat-evoked potential
CPM: Conditional pain modulation
CPT: Cold pain threshold
DLPFC: Dorsolateral prefrontal cortex
ECOG: Eastern Cooperative Oncology Group
EEG: Electroencephalography
EOC: Ente Ospedaliero Cantonale
EQ-5D-3 L: European Quality of Life 5 Dimensions 3 Level Version
GAD: Generalized Anxiety Disorder scale
HPT: Heat pain threshold
M1: Primary motor cortex
MFIS: Modified Fatigue Impact Scale
MOS: Medical Outcomes Study
NRS: Numerical rating scale
OA: Offset analgesia
OFQ: Oncofog Questionnaire
PHQ: Patient Health Questionnaire
QST: Quantitative sensory testing
RCT: Randomised clinical trial
SERGAS-FBGS: Servizo Galego de Saúde - Fundación Biomédica Galicia Sur
SERGAS-IDIS: Servizo Galego de Saúde - Instituto de Investigación Sanitaria de Santiago de Compostela
SF-36: 36-item short survey
tACS: transcranial alternating current stimulation
tDCS: transcranial direct stimulation
TECHNION: Israel Institute of Technology
tES: transcranial electrical stimulation
TSP: Temporal summation of pain
UMG: Universitätsmedizin Göttingen
USC: Universidade de Santiago de Compostela
ZBI: Zarit Burden Interview
